# Web App for prediction of hospitalisation in Intensive Care Unit by covid-19

**DOI:** 10.1590/0034-7167-2022-0740

**Published:** 2023-12-04

**Authors:** Greici Capellari Fabrizzio, Alacoque Lorenzini Erdmann, Lincoln Moura de Oliveira

**Affiliations:** I Universidade Federal de Santa Catarina. Florianópolis, Santa Catarina, Brazil; II Universidade Federal do Ceará. Fortaleza, Ceará, Brazil

**Keywords:** Inventions, Forecasting, Artificial Intelligence, Covid-19, Precision Medicine, Invenciones, Predicción, Intelligence Artificielle, Covid-19, Médecine de Précision, Invenções, Predição, Inteligência Artificial, Covid-19, Medicina de Precisão

## Abstract

**Objective:**

To develop a Web App from a predictive model to estimate the risk of Intensive Care Unit (ICU) admission for patients with covid-19.

**Methods:**

An applied technological production research was carried out with the development of Streamlit using Python, considering the decision tree model that presented the best performance (AUC 0.668).

**Results:**

Based on the variables associated with Precision Nursing, Streamlit stratifies patients admitted to clinical units who are most likely to be admitted to the Intensive Care Unit, serving as a decision-making support tool for healthcare professionals.

**Final considerations:**

The performance of the model may have been influenced by the start of vaccination during the data collection period, however, the Web App via Streamlit proved to be a feasible tool for presenting research results, due to the ease of understanding by nurses and its potential for supporting clinical decision-making.

## RESUMO

**Objetivo:**

Desenvolver um *Web App* a partir de um modelo preditivo para estimar o risco de internação de pacientes com covid-19 em UTI.

**Métodos:**

Realizou-se uma pesquisa aplicada de produção tecnológica com o desenvolvimento do *Streamlit* a partir do Python, considerando o modelo de árvore de decisão que apresentou o melhor desempenho (AUC 0.668).

**Resultados:**

A partir das variáveis associadas à Enfermagem de Precisão, o *Streamlit* estratifica os pacientes internados nas unidades clínicas com maior probabilidade de internação em Unidade de Terapia Intensiva, funcionando como uma ferramenta de apoio à tomada de decisão dos profissionais de saúde.

**Considerações finais:**

A performance do modelo pode ter sido influenciada pelo início da vacinação no período de coleta de dados, no entanto, o *Web App* via *Streamlit* mostrou-se uma ferramenta viável para a apresentação dos resultados de pesquisa, devido à facilidade de entendimento por parte dos enfermeiros e pelo potencial de apoio à decisão clínica.

## RESUMEN

**Objetivo:**

Desarrollar una Web App a partir de un modelo predictivo para estimar el riesgo de ingreso a la Unidad de Cuidados Intensivos (UCI) para pacientes con covid-19.

**Métodos:**

Se realizó una investigación de producción tecnológica aplicada con el desarrollo de Streamlit utilizando Python, considerando el modelo de árbol de decisiones que presentó el mejor rendimiento (AUC 0.668).

**Resultados:**

Basado en las variables asociadas con la Enfermería de Precisión, Streamlit estratifica a los pacientes ingresados en unidades clínicas que tienen más probabilidades de ser admitidos en la Unidad de Cuidados Intensivos, sirviendo como una herramienta de apoyo para la toma de decisiones para los profesionales de la salud.

**Consideraciones finales:**

El rendimiento del modelo puede haber sido influenciado por el inicio de la vacunación durante el período de recolección de datos. La Web App a través de Streamlit demostró ser una herramienta factible para presentar los resultados, debido a la facilidad de comprensión y su potencial para apoyar la toma de decisiones clínicas.

## INTRODUCTION

Patients infected with covid-19 exhibit a varied clinical progression of the disease, with signs and symptoms ranging from mild conditions to moderate and severe forms of infection. Among patients who develop moderate cases, 20 to 30% require care in a hospital clinical bed, and 5 to 25% of patients who develop severe cases require care in an Intensive Care Unit (ICU)^(1-2)^.

Several studies propose the development of artificial intelligence models to predict the likelihood of covid-19 infected patients experiencing a worsening clinical condition and requiring ICU admission^(1-3)^. These technologies provide accurate statistical predictions that assist in healthcare professionals’ decision-making process^(4)^. However, one of the challenges lies in making research results available so that healthcare professionals can utilize them. One tool we found for this purpose was Streamlit, a Web App that uses an open-source Python library to facilitate the creation and sharing of custom web applications, leveraging machine learning and data science^(5)^. No reports in the scientific literature describe applications for presenting the results of scientific research, where a web calculator allows healthcare professionals to carry out simulations to estimate the risk of hospitalization for patients with covid-19 in the ICU.

These artificial intelligence techniques contribute to improvements in Nursing care and health care as a whole, using Precision Nursing as a theoretical framework that assists providers in customizing care^(6)^. Precision Nursing, a concept derived from Precision Medicine, is characterized as nursing care tailored to a patient’s unique characteristics and needs, taking into account biological specificities, clinical and laboratory biomarkers, underlying pathologies, lifestyle habits (phenotype), social determinants, and the context in which the patient is situated (epigenetics)^(6-8)^. The identification of biomarkers broadens the opportunities for nurses to identify which underlying biological mechanisms are behind a specific health condition or symptom, thereby applying more precise interventions for that individual’s health recovery^(7)^.

The use of precision tools aids healthcare professionals in stratifying patients who have a higher need for personalized care, as well as identifying patients with a higher risk and, therefore, who require a distinct follow-up schedule. Another potentially useful aspect is in guiding discussions with families about long-term outcomes, such as in Post-covid-19 Syndrome or Long covid^(9-10)^.

## OBJECTIVE

To develop a Web App from a predictive model to estimate the risk of Intensive Care Unit (ICU) admission for patients with covid-19.

## METHODS

This applied research study focuses on the technological production of a Web App for predicting Intensive Care Unit (ICU) admissions due to covid-19. It was developed between November 2021 and March 2022. Applied research is characterized by its pursuit of immediate solutions to current problems^(11)^. Web Apps are computational tools ranging from simple web pages to comprehensive websites with specialized functionalities, capable of processing an array of information. These functionalities include the potential for database integration and the development of applications in specific areas^(12)^.

This study forms part of a doctoral thesis titled: “Precision Nursing and Artificial Intelligence: biomarkers for predicting the hospitalization of patients infected with covid-19 in an Intensive Care Unit”. The research was conducted under the Postgraduate Program in Nursing at the Federal University of Santa Catarina.

The production methodology for the Web App was divided into three stages:

### 1. Patient Information Recruitment:

The study involved 547 patients diagnosed with covid-19, admitted to five Brazilian university hospitals: one in the South, two in the Southeast, one in the Northeast, and one in the North.

Data were collected through telephone interviews conducted from April to December 2021, based on a list provided by the university hospitals. During the initial contact, patients were invited to participate in the study after the Informed Consent Form was read to them. Upon agreement, the interview was recorded, and two questionnaires were administered - the Care Transitions Measure (CTM-15), which evaluates the transition from hospital care to home care, and the Patient Measure Of Safety (PMOS), which assesses patient safety in the hospital context. Next, a structured sociodemographic questionnaire was used to profile the patients. All responses were recorded via the Google Forms® platform^(13-14)^.

The selection criteria included a positive test for SARS-CoV-2 and hospital admission lasting longer than 72 hours. If patients were debilitated or experienced physical, psychological, and cognitive changes, a responsible party, family member, or caregiver was invited to respond, as the CTM-15 allows responses from a caregiver or responsible party. However, patients with debilitated conditions or psychological incapacity to respond to the survey were excluded from the study, as the PMOS must be completed by the patient who experienced the situations influencing their safety. Also excluded were minors, patients who could not communicate fluently in Portuguese, patients not discharged to their homes, patients who died after discharge, and patients readmitted after discharge.

We analyzed 23 variables associated with Precision Nursing, including: patient sociodemographic information (age, gender, level of education, race); clinical biomarkers (days of ICU admission, chronic respiratory disease, systemic arterial hypertension, cardiovascular diseases, diabetes mellitus, kidney diseases, obesity, cancer, fever, fatigue, shortness of breath, cough, loss of smell and taste, headache, body aches, nausea, vomiting, and diarrhea); lifestyle habits (phenotype) such as smoking history; and social determinants and context (epigenetics), like family income and the number of people living in the household. The excluded variables were: place of residence, days of hospitalization, and use of mechanical ventilation, either because they could reveal the outcome or did not contribute to the modeling.

Standardization of numerical variables (age and number of people living in the household) was achieved by removing the mean and scaling around unit variance. Other variables were considered categorical and transformed into binary values of 0 or 1 using the one-hot encoding technique, allowing data to be modeled numerically. After these standardization techniques were applied, missing values were filled to complete the database.

The database comprised 309 patients who had been in the ICU and 238 who had not. This discrepancy between classes could potentially facilitate learning of the class with more records. To balance the classes, the undersampling technique was employed. Data from both classes were mixed through random selection, and a new grouping was made based on the class with the fewer records, resulting in 476 patients.

### Model Training and Validation

The data were randomly divided, with 75% (357 patients) allocated for model training and 25% (119 patients) for testing. We tested the following artificial intelligence models: neural network, AdaBoost, logistic regression, random forest, K Nearest Neighbor, Naive Bayes, Support Vector Machine, and decision tree. These eight models underwent evaluation through a five-fold cross-validation process, using metrics such as Area Under the Curve (AUC), sensitivity, and specificity. The k-fold technique, previously described for five folds, involves random division of the sample into a specific number of iterations and groups-in this case, five of each. In each iteration, four groups are designated for training and one for testing. In the next iteration, these groups are changed, and the final accuracy is computed from the mean accuracy of the iterations^(15)^. The AUC metric refers to the largest area under the Receiver Operating Characteristic (ROC) curve and correlates with the sensitivity and specificity measurements provided by the classifier. These ranged from 0.585 to 0.668, with the decision tree model demonstrating the highest performance (AUC 0.668)^(15)^. The three most influential variables in the model’s decision were age, admission to the Federal University of Amazonas hospital, and the number of people living in the same household, followed by other variables.

Sensitivity is assessed by calculating the number of patients classified as true positives, divided by the sum of true positives and false negatives, thereby gauging the model’s positive result percentage. Conversely, specificity is evaluated by calculating the number of patients classified as true negatives divided by the sum of true negatives and false positives. This measures the model’s capacity to detect negative results^(15)^.

The confusion matrix generated from the decision tree model’s test data revealed that the model classified 119 patients (25% of the database) into each of the classes. Of these, 32.77% (39 patients) were classified as true positives, 31.93% (38 patients) as true negatives, 18.49% (22 patients) as false positives, and 16.81% (20 patients) as false negatives. For this model, efforts were made to minimize the number of patients classified as false negatives-that is, patients who require ICU admission yet were classified by the model as unlikely to need ICU care, given the practical implications of this classification.

### Web App Development

The Web App development team consisted of a multidisciplinary trio: a software engineer and two nurses, all specialists in health management, with one also expert in health informatics. The nurses played key roles in data collection, defining study variables, and selecting the most appropriate metrics for model evaluation. The developer coded the source in Python, facilitating variable standardization and the training and testing of models, which led to the subsequent development of Streamlit. Additionally, the developer created the GitHub repository.

**Figure 1:**
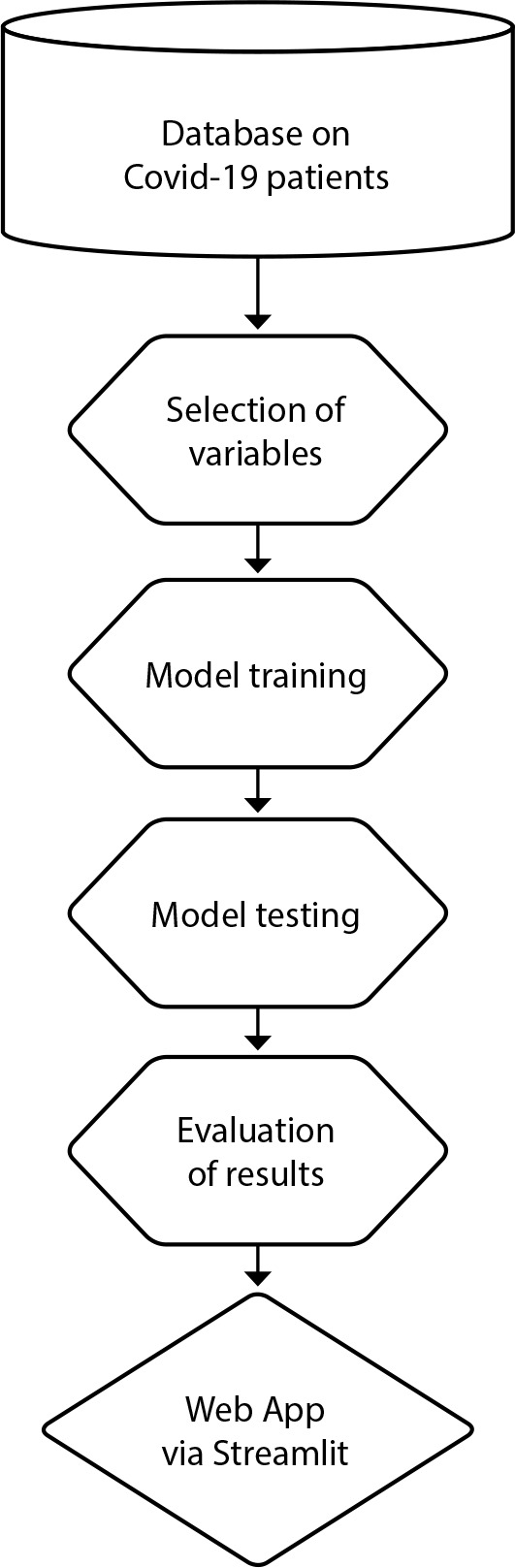
Flowchart of the Web App Construction, Florianópolis, Santa Catarina, Brazil, 2022

The Web App’s construction flowchart, illustrated in Figure 1, traces the journey from the raw database, through the training and testing of models, to the final construction of the Web App.

The project was developed using the Python programming language, wherein the best models were identified, followed by the execution of Streamlit. Initially, the ‘streamlit run’ command was carried out in a Python script, which activated a local Streamlit server, and the application was opened in a new tab of the default web browser.

The application can be customized with text, graphics, figures, tables, and other necessary elements to tailor the application according to the requirements. These changes can be implemented by modifying the source code and saving the source file. Streamlit detects these changes and prompts the user to apply them, enabling swift interaction between the ongoing code development and the visualization of the application^(5)^.

The data is stored on GitHub in a Microsoft Office Excel spreadsheet and is accessed by Streamlit. The data updates occur by updating the Excel spreadsheet, at which point the tool queries the file and updates its outputs (predictions) for new data entries (new patient).

The study was conducted in accordance with national and international ethical guidelines and received approval from the Research Ethics Committee of the Federal University of Santa Catarina. Concerning the ethical principles of research involving human subjects, the study participants were presented with an Informed Consent Form, which was signed by all participants. Moreover, the study adhered to the guidance of the Transparent Reporting of a Multivariable Prediction Model for Individual Prognosis or Diagnosis (TRIPOD) for reporting research on the development, validation, or updating of prediction models for diagnostic or prognostic purposes^(16)^.

## RESULTS

The Web App was developed using a decision tree, which stood out as the most effective artificial intelligence model. The description of the code and the execution of Streamlit were performed in Python.

**Figure 2:**
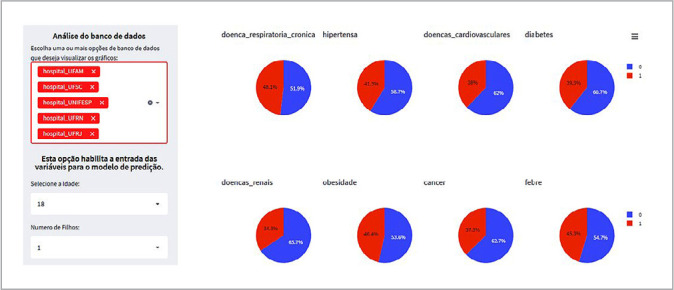
Visual and interactive exploration of the database in the Web App, Florianópolis, Santa Catarina, Brazil, 2022

As illustrated in Figure 2, the user interface allows for a visual and interactive display of variables related to Precision Nursing. For instance, signs and symptoms of covid-19, demographic information, and patients’ prior illnesses in the database can be viewed. This data exploration enables nurses to understand the characteristics of the patients involved in the study. Additionally, traits specific to a hospital or even multiple hospitals can be explored based on the command chosen by the user.

Let’s consider a practical application of the tool. A nurse in the covid-19 ward receives a 37-year-old white male patient with a completed higher education, two children, and a family income between R$ 5,000 and R$ 10,000. He has no comorbidities and presents the following symptoms: shortness of breath, cough, and fever. The nurse aims to determine the severity of the patient’s condition to plan appropriate nursing care and, if necessary, request an ICU bed. After collecting the patient’s information, the nurse enters the variables into the Web App and views a prediction of the probability of the patient’s admission to the ICU.

**Figure 3:**
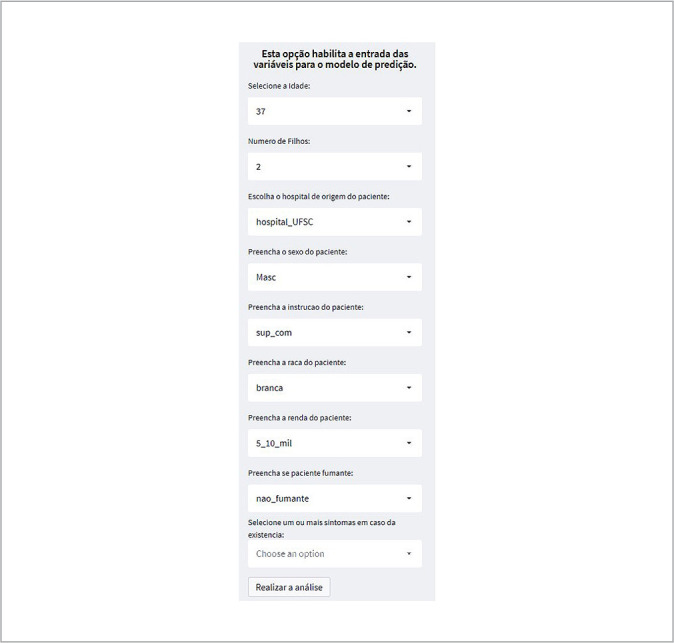
Input of patient’s clinical data into the Web App, Florianópolis, Santa Catarina, Brazil, 2022

As illustrated in the previous example, the nurse can use the Web App as a decision support tool. In the application’s left-hand menu, variables associated with Precision Nursing can be entered, representing the clinical profile of a newly admitted patient, as shown in Figure 3.

**Figure 4:**
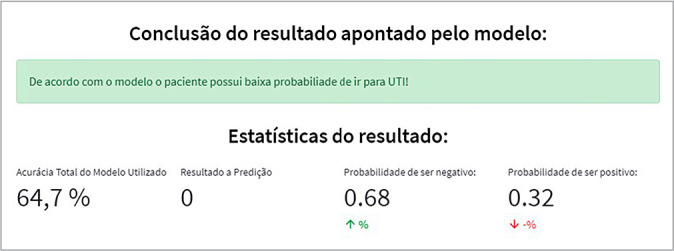
Simulation of a case where the patient has a low probability of requiring ICU admission, Florianópolis, Santa Catarina, Brazil, 2022

Upon entry of the patient’s clinical data, the prediction is processed using an artificial intelligence model trained with previous patients’ data. This model determines the likelihood of the new patient requiring ICU admission or not. Figure 4 displays a result indicating a 68% probability that the patient will not require ICU admission, with a model accuracy of 64.7%.

**Figure 5:**
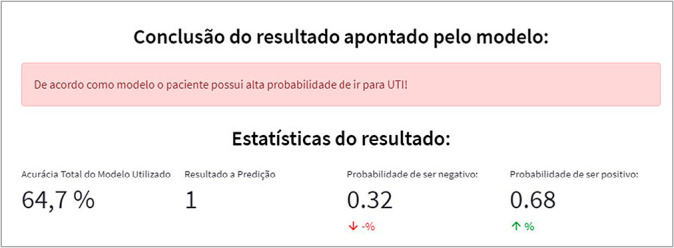
Simulation of a case where the patient has a high probability of requiring ICU admission, Florianópolis, Santa Catarina, Brazil, 2022

In contrast, Figure 5 presents a prediction model classification where the patient has a 68% likelihood of needing ICU admission (class 1). In classifications where the result suggests a high probability of ICU admission, the result is displayed on a color scale ranging from green (low probability) to red (high probability).

Beyond predicting the likelihood of ICU admission, the tool also displays the variables that most influenced the model and the confusion matrix, after prediction.

The development of this technological innovation proved to be an efficient way of presenting research results stemming from a doctoral thesis. It demonstrated the potential of the Web App, built via Streamlit, to predict the likelihood of ICU admission for patients infected with covid-19, based on the theoretical framework of Precision Nursing. Apart from risk stratification features for ICU admission, the tool also showed potential for storing information related to simulations performed in the Web App, which can be leveraged in the future to enhance the model’s predictive performance. The recording of this information can track changes in the disease’s behavior over time.

## DISCUSSION

Of the eight models tested, the decision tree delivered the best AUC (0.668). This result was achieved with the following parameters: a binary tree was induced, the minimum number of cases in branches was set to 5, and the maximum depth of the trees was set at 12. Moreover, it was parameterized not to split subsets smaller than 7 and to halt when the majority reached 99%. Setting the maximum depth of the tree to 12 helps prevent overfitting, i.e., the over-adjustment that can occur in trees with many branches.

A decision tree is characterized by nodes (leaves) and branches. Each node represents a test performed on an attribute, with the branches being the results of these tests. Thus, some data follow one branch while the rest follow another. Therefore, each subsequent node receives fewer samples than the previous node^(17)^. The decision tree is among the most elementary machine learning algorithms, facilitating understanding for healthcare professionals as the interpretability and explainability of the model are evident. This makes it clear how the model classifies patients.

It is hypothesized that the performance of the decision tree model (AUC 0.668) may be related to a crucial variable not considered in this study: the patients’ vaccination status. We know that vaccination reduces severe cases of covid-19, potentially leading to a reduction of 65.6% in ICU admissions among vaccinated patients^(18)^. For those who are fully vaccinated, the probability of ICU admission drops by 48.8%, the likelihood of needing a mechanical ventilator decreases even more significantly, dropping to 55.4%, and they have a 22.6% lower risk of death. Vaccinated patients typically have shorter hospital stays, while unvaccinated patients tend to have longer periods off work^(19)^.

In regards to Streamlit, the chosen Web App for the study, previous predictive studies in the healthcare field have used Streamlit as a tool to make the optimal artificial intelligence model accessible for professionals. A retrospective study conducted in China included 385 participants for predicting the risk of leakage from injected liquid polymethylmethacrylate in patients with osteoporotic vertebral compression fractures undergoing percutaneous vertebroplasty. Using clinical variables, surgical details, and baseline characteristics of hospitalized patients with or without leakage, they developed an artificial intelligence model to assess the risk of potential postoperative leakage in patients undergoing percutaneous vertebroplasty^(20)^.

Another retrospective cohort study included 103 boys diagnosed with a posterior urethral valve. The patients were tracked for 5.7 years, with the hypothesis that early renal and anatomical characteristics could be predictive of future progression to chronic kidney disease, renal replacement therapy, and intermittent catheterization^(9)^. The application was cited as a simple, easy-to-use, and intuitive tool for nurses and other healthcare professionals^(4)^. Mobile applications, like this Web App, create a connection between a mobile device and web-based content through a specially-designed browser for the specific purpose of predicting the admission of patients infected with covid-19 to the Intensive Care Unit^(12)^.

However, when creating a predictive Web App for clinical use, care must be taken to not demand too much information (variables) from patients, as it isn’t feasible for professionals to input a large number of variables in situations that require swift decision-making by the team^(3)^. To address this, we considered 23 predictive variables, which can be swiftly gathered in clinical practice.

The development of this application was enabled by merging knowledge from the fields of engineering and health. Specifically, we relied on the expertise of nurses who provided scientific, theoretical, and practical insights related to healthcare, to design the application with a software engineer responsible for code development. This blend of knowledge directs the project, fostering the convergence of healthcare professionals’ needs and technological possibilities. Such strategies encourage the adoption of the tool in nursing practice^(4)^.

In this context, the Web App offers the potential to develop software-building skills among nursing and health professionals. As they begin using it, it becomes a valuable asset in clinical practice, assisting healthcare professionals in decision-making. On the other hand, the challenges associated with constructing such technology may lie in the need for professional training in digital health and potential resistance from health professionals themselves in undertaking this type of project.

## FINAL CONSIDERATIONS

The decision tree algorithm provided the highest AUC (0.668) for predicting the hospitalization of covid-19 infected patients in the Intensive Care Unit. This was the rationale behind its selection as the model to be uploaded to Streamlit. The performance of the model may have been influenced by changes in the disease’s behavior, such as the emergence of new variants with distinct predictive variables and the initiation of vaccination during the period of data collection. Therefore, new studies that analyze these variables are recommended.

The Web App via Streamlit simplifies the creation and operation of tools compared to other methods, enhancing the understanding of healthcare professionals and demonstrating its potential as a clinical decision-making support tool. This might inspire professionals in health and technology fields to develop similar solutions tailored to their specific needs and areas of expertise. In this context, interdisciplinarity was a cornerstone of the study: a team composed of nurses and a software engineer was formed, allowing for a fusion of skills. Each professional brought their own expertise to the collaborative construction of the tool.

Furthermore, it is possible to store simulations carried out in the calculator for subsequent use to improve the predictive performance of the model. New data can capture changes in behavior over time, influencing the response of the variables and, consequently, the model’s performance.

The study does exhibit certain limitations, particularly in terms of the need for tool validation by users, patients, health professionals, and administrators. It is also recommended that the project be continued to ensure the web application becomes interoperable with the patient’s electronic health record.
